# Determinants of linear growth faltering among children with moderate-to-severe diarrhea in the Global Enteric Multicenter Study

**DOI:** 10.1186/s12916-019-1441-3

**Published:** 2019-11-25

**Authors:** Rebecca L. Brander, Patricia B. Pavlinac, Judd L. Walson, Grace C. John-Stewart, Marcia R. Weaver, Abu S. G. Faruque, Anita K. M. Zaidi, Dipika Sur, Samba O. Sow, M. Jahangir Hossain, Pedro L. Alonso, Robert F. Breiman, Dilruba Nasrin, James P. Nataro, Myron M. Levine, Karen L. Kotloff

**Affiliations:** 1 Department of Epidemiology, University of Washington, Seattle, WA USA; 2 Department of Global Health, University of Washington, Seattle, WA USA; 3 Department of Epidemiology, Global Health, Pediatrics, Medicine, Childhood Acute Illness and Nutrition Network, University of Washington, Seattle, WA USA; 4 Department of Epidemiology, Global Health, Pediatrics, Medicine, University of Washington, Seattle, WA USA; 5 Department of Global Health, Health Services, Health Metrics Sciences, University of Washington, Seattle, WA USA; 6 International Centre for Diarrhoeal Disease Research, Mohakhali, Dhaka, Bangladesh; 7 Department of Pediatrics and Child Health, Aga Khan University, Karachi, Pakistan; 8 Present Address: Enteric and Diarrheal Diseases Program, Bill and Melinda Gates Foundation, Seattle, WA USA; 9 National Institute of Cholera and Enteric Diseases, Kolkata, India; 10 Present Address: Translational Health Science and Technology Institute, Faridabad, India; 11 Centre pour le Développement des Vaccines, Bamako, Mali; 12 Medical Research Council Unit, The Gambia at London School of Hygiene & Tropical Medicine, Banjul, The Gambia; 13 Centro de Investigação em Saúde da Manhiça, Maputo, Mozambique; 14 Present Address: Global Malaria Programme, World Health Organization, Geneva, Switzerland; 15 Global Disease Detection Division, Kenya Office of the US Centers for Disease Control and Prevention, Nairobi, Kenya; 16 Present Address: Global Health Institute Emory University, Atlanta, GA USA; 17 Center for Vaccine Development, Department of Medicine, University of Maryland School of Medicine, Baltimore, MD USA; 18 Center for Vaccine Development, Department of Medicine, Department of Pediatrics, University of Maryland School of Medicine, Baltimore, MD USA; 19 Present Address: Department of Pediatrics, University of Virginia School of Medicine, Charlottesville, VA USA; 20 Center for Vaccine Development and Global Health, Department of Pediatrics and Medicine, Department of Pediatrics, University of Maryland School of Medicine, Baltimore, MD USA; 21 Center for Vaccine Development and Global Health, Department of Pediatrics and Medicine, University of Maryland School of Medicine, Baltimore, MD USA

**Keywords:** Diarrheal diseases, Malnutrition, Stunting, Growth retardation, Nutritional deterioration, Diarrhea sequelae, Clinical prediction

## Abstract

**Background:**

Moderate-to-severe diarrhea (MSD) in the first 2 years of life can impair linear growth. We sought to determine risk factors for linear growth faltering and to build a clinical prediction tool to identify children most likely to experience growth faltering following an episode of MSD.

**Methods:**

Using data from the Global Enteric Multicenter Study of children 0–23 months old presenting with MSD in Africa and Asia, we performed log-binomial regression to determine clinical and sociodemographic factors associated with severe linear growth faltering (loss of ≥ 0.5 length-for-age *z*-score [LAZ]). Linear regression was used to estimate associations with ΔLAZ. A clinical prediction tool was developed using backward elimination of potential variables, and Akaike Information Criterion to select the best fit model.

**Results:**

Of the 5902 included children, mean age was 10 months and 43.2% were female. Over the 50–90-day follow-up period, 24.2% of children had severe linear growth faltering and the mean ΔLAZ over follow-up was − 0.17 (standard deviation [SD] 0.54). After adjustment for age, baseline LAZ, and site, several factors were associated with decline in LAZ: young age, acute malnutrition, hospitalization at presentation, non-dysenteric diarrhea, unimproved sanitation, lower wealth, fever, co-morbidity, or an IMCI danger sign. Compared to children 12–23 months old, those 0–6 months were more likely to experience severe linear growth faltering (adjusted prevalence ratio [aPR] 1.97 [95% CI 1.70, 2.28]), as were children 6–12 months of age (aPR 1.72 [95% CI 1.51, 1.95]). A prediction model that included age, wasting, stunting, presentation with fever, and presentation with an IMCI danger sign had an area under the ROC (AUC) of 0.67 (95% CI 0.64, 0.69). Risk scores ranged from 0 to 37, and a cut-off of 21 maximized sensitivity (60.7%) and specificity (63.5%).

**Conclusion:**

Younger age, acute malnutrition, MSD severity, and sociodemographic factors were associated with short-term linear growth deterioration following MSD. Data routinely obtained at MSD may be useful to predict children at risk for growth deterioration who would benefit from interventions.

## Background

Chronic malnutrition is highly prevalent among children under age 5 globally, with the greatest burden affecting children in low- and middle-income countries (LMICs) in Africa and Asia [1]. Stunting, defined as height- or length-for-age (HAZ/LAZ) less than 2 standard deviations below the population standard mean [2], is an indicator of chronic malnutrition [3]. Fifteen percent of all deaths and 21% of disability-adjusted-life-years in children under 5 years have been attributed to stunting [4]. Stunting also has long-term consequences, including impaired cognitive development, increased risk of non-communicable disease in adulthood, and decreased economic productivity [5].

Although the etiology of chronic malnutrition is multi-faceted, an estimated 13.5% of global stunting prevalence is attributable to diarrheal disease [6]. A meta-analysis of longitudinal studies in 5 LMICs reported a child’s odds of stunting at 24 months of age increased by 16% with every 5% increase in incidence of diarrhea (odds ratio 1.16 [95% confidence interval (95% CI) 1.07, 1.25]) [7]. In addition, children in seven LMICs across Africa and Asia who experienced moderate-to-severe diarrhea (MSD) lost significantly more height/length for age *z*-score (HAZ/LAZ) in the 2–3 months following the episode than age- and village-matched controls [8].

Addressing linear growth faltering in children with MSD may be an important step towards reducing stunting and its long-term consequences. This may be particularly true for those under 24 months of age, as this is the critical time period in which most growth faltering occurs [9] and during which interventions are likely to be effective. However, it is unclear which groups of children are at highest risk. In addition, few interventions have been successful at mitigating the nutritional consequences of diarrhea [10]. Identifying risk factors for post-MSD linear growth faltering can inform which groups of children should be prioritized for inclusion in trials of potential interventions, and, once an effective intervention has been identified, to optimize the effectiveness of intervention delivery within programs by targeting children at high risk of growth faltering.

Using data from children under 24 months old with MSD enrolled in a previous large diarrhea etiology study (the Global Enteric Multicenter Study, or GEMS), we sought to identify determinants of linear growth faltering in the 60–90 days following presentation with MSD. We evaluated the frequency and severity of linear growth faltering in this population and identified the clinical, host, and socioeconomic factors associated with faltering in linear growth during the short-term follow-up period. We also developed and validated a predictive model and risk scoring tool for estimating an individual child’s risk of short-term growth faltering following MSD.

## Methods

### Study setting and populations

GEMS [8] was a large case-control study of the incidence, etiology, and clinical consequences of MSD among children 0–59 months of age conducted between 2007 and 2011 in Bangladesh, India, Pakistan, Kenya, Mali, Mozambique, and The Gambia. Here we describe a case-only analysis, using data on MSD cases in GEMS, defined as children seeking care at study health facilities for an episode of new (onset after ≥ 7 diarrhea-free days) and acute diarrhea (≥ 3 abnormally loose stools within the previous 24 h with an onset within the previous 7 days) with at least one of the following characteristics: dehydration (presence of sunken eyes, loss of skin turgor, intravenous hydration administered or prescribed), dysentery (presence of visible blood in diarrhea), or clinical decision to admit to hospital. Children presenting with prolonged (> 7 days’ duration) and persistent (> 14 days’ duration) diarrhea were excluded. GEMS included a single follow-up visit predefined at 60 days (with an acceptable range of 50–90 days) following enrollment. Study clinicians performed physical exams and conducted interviews with caregivers at enrollment and at follow-up to ascertain clinical, anthropometric, and sociodemographic factors. Children’s weight was measured at enrollment (MSD presentation). Child’s length and middle-upper arm circumference (MUAC) were measured 3 times at each visit, and median measures used in the analysis. Study clinicians also abstracted data from medical records if the child was hospitalized at enrollment. The clinical and epidemiological methods used in GEMS, including the standardized procedures for obtaining anthropometric measurements, have been described in detail [11].

This post hoc analysis used the enrollment and follow-up data of the MSD cases enrolled in GEMS, restricting to children under 24 months of age. Children were therefore included in this analysis if they were an MSD case, were under 24 months of age, and had both LAZ measurements available at enrollment and follow-up; therefore, children who died or were lost to follow-up were excluded. We also excluded children with implausible length/LAZ values (LAZ > 6 or < − 6 and change in (Δ) LAZ > 3; a length gain of > 8 cm for follow-up periods 49–60 days and > 10 cm for periods 61–91 days among infants ≤ 6 months, a length gain of > 4 cm for follow-up periods 49–60 days and > 6 cm for periods 61–91 days among children > 6 months, or length values that were > 1.5 cm lower at follow-up than at enrollment). Because standards for MUAC are not available for children under 6 months of age, only MUAC measurements for children over 6 months of age were included in the analysis.

### Variables and definitions

#### Outcomes

We defined faltering in linear growth using change in length-for-age *z*-score (ΔLAZ) between enrollment and follow-up. Linear growth faltering was defined in two ways: (1) as a continuous variable (ΔLAZ) with ΔLAZ< 0 being considered a loss and (2) as a binary variable, severe linear growth faltering, defined as loss of 0.5 LAZ or more (ΔLAZ ≥ − 0.5).

#### Risk factors

Risk factors examined in this analysis included clinical and sociodemographic factors. Factors included age (per date of birth reported by the primary caretaker and verified by the child’s health card), sex, admission to hospital at presentation, presentation with fever (axillary temperature > 37.5 F), co-morbidities per final diagnosis indicated on medical records, LAZ at presentation calculated according to WHO standards [2], wasting (weight-for-length *z*-score [WLZ] < − 2 using WHO standards, using post-rehydration weight), dysentery (visible blood in stool observed by caregiver or health care provider at presentation), stunting (LAZ < − 2 using WHO standards), and duration of diarrhea (caregiver reported number of days the diarrhea has lasted at presentation). Anthropometric *z*-scores were calculated using WHO Stata macro code [12]. Duration of diarrhea was ascertained by summing the duration of diarrhea during the 7 days prior to enrollment (children with diarrhea lasting longer than 7 days were excluded from participation) plus duration of diarrhea during the 14 days after enrollment. Diarrhea duration for the 14 days following enrollment was ascertained using a memory aid suitable for groups of all literacy levels, which the caregiver returned at the follow-up visit, as depicted elsewhere [11]. Cessation of the enrollment episode was defined as two consecutive days in which diarrhea was not reported. Diarrhea was categorized as acute diarrhea (defined above), prolonged (> 7–13 days duration), or persistent (≥ 14 days duration). Sociodemographic characteristics were evaluated at enrollment and included access to improved water (caregiver report of the following: main source of drinking water for the household is piped into house or yard, public tap, tubewell, covered well, protected spring, rainwater, or borehole; is accessible within 15 min or less, roundtrip; and is available daily), access to improved defecation facility (caregiver report of access to the following: flush toilet, ventilated improved pit latrine with or without water seal, or pour flush toilet not shared with other households), caregiver handwashing (caregiver report of handwashing before eating, before handling child’s food, after defecation, or after disposing of child’s feces), and wealth quintile (quintile of a wealth effects score calculated from asset ownership information reported by caregiver at enrollment [13]). Caretakers were shown pictures to aid in accurate identification of water and sanitation facilities.

### Data analysis

#### Risk factor model

Univariate and multivariable relative risk regression models specifying a binomial distribution (or Poisson distribution if model failed to converge [14]) with robust standard errors were used to estimate relative risks of severe linear growth faltering and 95% confidence intervals (95% CIs). Univariate and multivariable linear regression models with robust standard errors were used to estimate continuous ΔLAZ and 95% CIs associated with the exposure variables of interest. Multivariable models were adjusted a priori for age, site, duration of follow-up, and LAZ at enrollment.

As children who were missing LAZ measurements at one or both of the study visits were excluded, we repeated the analysis of risk factors using imputed LAZ values for children in whom follow-up LAZ was missing due to loss to follow-up or death [15]. We conducted multiple imputation for monotone missing data, which assumes missingness at random conditional on observed characteristics. Imputation models included linear regression to impute ΔLAZ and Poisson regression to impute severe linear growth faltering. Variables were selected for inclusion in the imputation if they were associated with missingness, per *χ*^2^ tests for categorical variables and *t* tests for continuous variables. Diagnostics of the imputation models included examining imputed values for reasonableness (whether the values were plausible and scientifically sensible given the covariates in the model) and comparing distributions of imputed vs observed values. All analyses were conducted in Stata 14.

#### Clinical prediction tool

In addition to a risk factor model, a clinical prediction model was developed to identify the combinations of factors that best predicted a child’s risk of severe linear growth faltering in the 50–90 days following MSD. We included only the characteristics in Table 1 that are easily collectible in a clinical setting in the prediction model. The data were randomly divided into separate derivation and validation datasets of equal size, and *t* tests or *χ*^2^ tests used to identify differences in baseline characteristics between the datasets. A backward elimination approach [16, 17] was used to develop the model, in which all candidate variables are included and eliminated based on statistical significance (*p* ≤ 0.1). We used the Akaike Information Criterion (AIC), a measure of model fit that penalizes larger models and thus attempts to reduce overfitting, to select the best fit model. We translated the best-fit model into a practical risk scoring tool by assigning values for each predictor based on the beta-coefficients from the model as described elsewhere [18]. The sum of risk scores for each parameter was the total risk score for each child. To validate the model, the risk score was applied to the validation cohort, and AUC performance and Brier score were compared with the derivation cohort.

**Table 1:** Baseline characteristics of children with MSD included in this GEMS analysis

	*n* (%) or median (interquartile range)	
Sociodemographic characteristics		
Age, months	11	(7–16)
0–6 months	1077	(17.4%)
> 6–12 months	2361	(38.1%)
> 12–23 months	2765	(44.6%)
Site		
The Gambia	705	(11.4%)
Mali	1172	(18.9%)
Mozambique	410	(6.6%)
Kenya	961	(15.5%)
India	1195	(19.3%)
Bangladesh	993	(16.0%)
Pakistan	767	(12.4%)
Female	2681	(43.2%)
Access to improved water	2824	(45.5%)
Access to improved sanitation^a^	1153	(18.6%)
Wealth index score [13]	− 0.08	(− 0.71, 0.59)
Clinical characteristics at presentation		
Stunting	1478	(23.8%)
Wasting	1357	(21.9%)
Severe wasting^b^	470	(7.6%)
MUAC < 12.5 cm among 5126 children 6–23 months	863	(16.8% of 5126 children)
Fever	1788	(30.3%)
Current breastfeeding 1456 children < 6 months		
Exclusive	439	(30.2%)
Partial	951	(65.3%)
None	66	(4.5%)
Hospitalized at presentation	1229	(19.8%)
Dysentery at presentation^c^	1352	(21.8%)
≥ 1 IMCI general danger sign	3661	(59.0%)
Presented with at least 1 co-morbidity^d^	2076	(33.5%)
Pneumonia	420	(6.8%)
Malaria	1510	(24.3%)
Malnutrition	349	(5.6%)
Other invasive bacterial infection	82	(1.3%)
Upper respiratory tract infection	8	(0.1%)

^a^Flush toilet, ventilated improved pit latrine with or without water seal, or pour flush toilet not shared with other households

^b^Severe wasting defined as weight-for-length *z*-score < − 3

^c^Visible blood in stool observed by study staff or reported by caregiver at presentation; discharge diagnosis of dysentery per managing clinician upon leaving the healthcare facility; or observed in stool sample by laboratory staff

^d^Per discharge diagnoses documented on medical records

We assessed the ability of the risk score to discriminate between children with and without severe linear growth faltering, with risk score as the sole predictor, using receiver operating characteristic (ROC) analysis to calculate the area under the curve (AUC) [19]. We also estimated Brier scores to quantify the difference between the predicted and actual outcomes; useful prediction models have Brier scores < 0.25 [19]. Risk scores were dichotomized into the most predictive categories using the cut-point identified in ROC analysis, which optimizes sensitivity and specificity. Positive and negative predictive values (PPV, NPV) were also calculated.

## Results

**Fig. 1:**
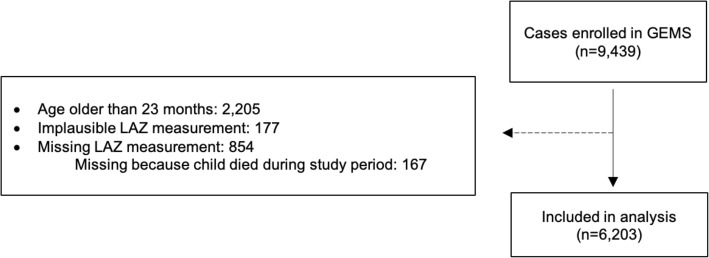
Flowchart of included subjects

Among the 9439 children with MSD who were enrolled in the GEMS study, 2205 children aged ≥ 24 months and 1031 children with a missing or implausible LAZ value were excluded. This resulted in 6203 surviving children under 24 months of age included in the analysis (Fig. 1). Median age of included children was 11 months (interquartile range 7–16) and 43.2% were female (Table 1). Distribution across the 7 sites was similar to that in the parent study: 705 (11.4%) in The Gambia, 1172 (18.9%) in Mali, 410 (6.6%) in Mozambique, 961 (15.5%) in Kenya, 1195 (19.3%) in India, 993 (16.0%) in Bangladesh, and 767 (12.4%) in Pakistan. Approximately 22% (*n* = 1352) of children presented with dysentery, 94.4% of whom were given or prescribed an antibiotic in the health facility (whereas 75.4% of children without dysentery were given an antibiotic). Thirty percent presented with fever and 19.8% were hospitalized at presentation. Approximately, one in four children presenting with MSD were stunted at presentation and one in five were wasted. Approximately 43.2% (*n* = 2681) of these children under 24 months of age experienced a subsequent diarrhea episode during the follow-up period, per caregiver report at the follow-up visit.

Mean ΔLAZ between enrollment and follow-up was − 0.25 (standard deviation [SD] 0.50). Median ΔLAZ was − 0.24 (interquartile range − 0.55, 0.05), and 28.6% developed severe linear growth faltering (loss of ≥ 0.5 LAZ) during the 90-day follow-up period. Notably, 82.9% of these children who lost ≥ 0.5 LAZ during follow-up were not stunted at MSD presentation, and 73.4% of these were not wasted. Children whose caregivers reported they experienced a subsequent diarrhea episode during follow-up lost slightly more LAZ (ΔLAZ = − 0.27) than those who did not (ΔLAZ − 0.23) (*p* value from *t* test = 0.01).

### Risk factor analysis

#### ΔLAZ

**Fig. 2:**
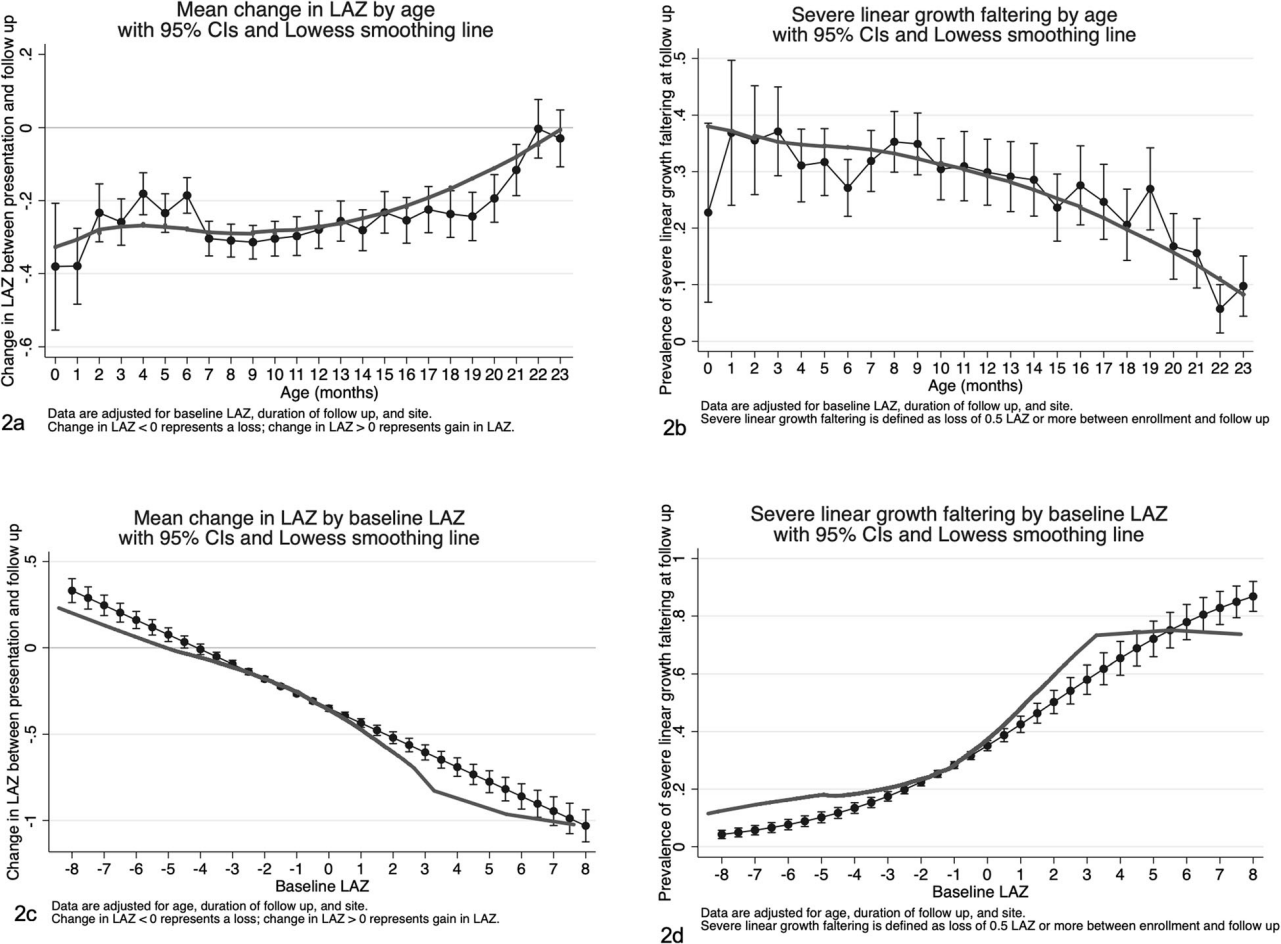
**a**–**d** Linear growth faltering following an episode of moderate-to-severe diarrhea by age and baseline LAZ

Age and nutritional status at MSD presentation, but not sex, were associated with ΔLAZ. Children > 6–12 months old lost approximately 0.07 more LAZ than children > 12–23 months (aβ − 0.10 [95% CI − 0.10, − 0.04]) adjusting for duration of follow-up, baseline LAZ, and site, and ΔLAZ was not statistically significantly different between children 0–6 months and those > 12–23 months (Table 2 and Fig. 2). Figure 2a depicts the pattern of ΔLAZ by age, demonstrating that the magnitude of LAZ loss decreased with each month gain in age. Children with higher baseline LAZ values experienced the greatest loss in LAZ (Fig. 2c), in an inverse relationship pattern; magnitude of LAZ loss decreased consistently with each unit increase in LAZ (aβ − 0.08 [95% CI − 0.09, − 0.07]). Children stunted at MSD presentation gained LAZ compared to their non-stunted counterparts (aβ 0.16 [95% CI 0.13, 0.19]) whereas wasted children lost an average of 0.21 LAZ more than children without wasting (95% CI − 0.24, − 0.18). Among children over 6 months of age, children with MUAC < 12.5 cm lost 0.12 more LAZ (95% CI − 0.15, − 0.08) than those with MUAC of ≥ 12.5 cm, after accounting for age, site, duration of follow-up, and baseline LAZ. Children who had a final diagnosis of malnutrition per discharge medical records lost 0.19 more LAZ than those who did not (95% CI − 0.24, − 0.13). Males’ ΔLAZ was similar to that of females (aβ 0.02 [95% CI − 0.0003, 0.05]).

**Table 2:** Risk factors for linear growth faltering among children 0–23 months old with MSD with complete outcome data. Statistically significant results (*p* < 0.05) are italicized. Asterisks (*) denote results from a robust Poisson model rather than log-binomial model

	ΔLAZ				Severe linear growth faltering			
	Mean	SD	Crude difference in change in LAZ	Adjusted for age, site, duration of follow-up, and baseline LAZ^a^	No. with outcome	Prevalence of loss of ≥ 0.5 LAZ	Crude relative risks	Adjusted for age, site, duration of follow-up, and baseline LAZ^a^
Age^b^								
0–6 months	− 0.27	0.70	*− 0.12 (− 0.14, − 0.09)*	− 0.02 (− 0.06, 0.01)	381	35.4%	*1.61 (1.45, 1.79)*	1.41 (1.22, 1.51)*
> 6–12 months	− 0.31	0.49	*− 0.08 (− 0.11, − 0.04)*	*− 0.07 (− 0.10, − 0.04)*	788	33.4%	*1.52 (1.39, 1.66)*	1.36 (1.22, 1.51)*
> 12–23 months	− 0.19	0.40	Reference	Reference	607	22.0%	Reference	Reference
Stunting^c^								
No	− 0.29	0.49	Reference	Reference	1472	31.2%	Reference	Reference
Yes	− 0.12	0.50	*0.17 (0.14, 0.20)*	*0.16 (0.13, 0.19)*	304	20.6%	*0.66 (0.59, 0.74)*	0.69 (0.61, 0.78)*
Sex								
Male	− 0.25	0.53	Reference	Reference	847	19.6%	Reference	Reference
Female	− 0.24	0.47	0.01 (− 0.01, 0.04)	0.02 (− 0.0003, 0.05)	588	17.7%	*0.92 (0.85, 1.00)*	0.91 (0.83, 1.00)*
Wasting								
No	− 0.22	0.51	Reference	Reference	1303	26.9%	Reference	Reference
Yes	− 0.34	0.48	*− 0.12 (− 0.15, − 0.09)*	*− 0.21 (− 0.24, − 0.18)*	473	34.9%	*1.30 (1.19, 1.41)*	*1.57 (1.41, 1.76)*
MUAC (among 6–23 months)								
**≥** 12.5	− 0.24	0.45	Reference	Reference	1129	26.5%	Reference	Reference
< 12.5 cm	− 0.27	0.46	*− 0.04 (− 0.07, − 0.004)*	*− 0.12 (− 0.15, − 0.08)*	266	30.8%	*1.16 (1.02, 1.33)*	*1.42 (1.23, 1.64)*
Current breastfeeding (among < 6 months)								
Exclusive	− 0.30	0.73	Reference	Reference	145	33.0%	Reference	Reference
Partial	− 0.25	0.68	0.05 (− 0.05, 0.14)	− 0.01 (− 0.10, 0.09)	323	34.0%	1.03 (0.88, 1.21)	1.12 (0.90, 1.40)*
None	− 0.28	0.75	0.01 (− 0.20, 0.23)	− 0.11 (− 0.32, 0.11)	25	37.9%	1.15 (0.82, 1.61)	1.41 (0.91, 2.20)*
Diarrhea type^d^								
Acute	− 0.23	0.48	Reference	Reference	829	26.6%	Reference	Reference
Prolonged	− 0.26	0.50	*− 0.01 (− 0.07, − 0.003)*	− 0.01 (− 0.04, 0.01)	568	29.8%	1.12 (1.01, 1.25)	1.08 (0.97, 1.20)*
Persistent	− 0.26	0.50	− 0.03 (− 0.06, 0.01)	− 0.003 (− 0.04, 0.04)	196	29.9%	1.12 (0.96, 1.31)	0.98 (0.95, 1.30)*
Hospitalized at enrollment								
No	− 0.23	0.49	Reference	Reference	1386	21.0%	Reference	Reference
Yes	− 0.32	0.43	*− 0.09 (− 0.12, − 0.06)*	*− 0.11 (− 0.14, − 0.07)*	491	31.7%	*1.37 (1.26, 1.49)*	1.36 (1.20, 1.53)*
Presentation with fever								
No	− 0.22	0.51	Reference	Reference	1074	26.1%	Reference	Reference
Yes	− 0.31	0.49	*− 0.10 (− 0.13, − 0.07)*	*− 0.09 (− 0.11, − 0.06)*	616	34.5%	*1.32 (1.22, 1.43)*	1.25 (1.12, 1.38)*
Presentation with dysentery								
No	− 0.26	0.51	Reference	Reference	1117	24.7%	Reference	Reference
Yes	− 0.20	0.49	*0.06 (0.03, 0.9)*	*0.07 (0.04, 0.11)*	310	22.6%	0.91 (0.82, 1.02)	0.88 (0.75, 1.02)*
Co-morbidities								
None	− 0.23	0.49	Reference	Reference	1090	26.4%	Reference	Reference
Any	− 0.28	0.51	*− 0.06 (− 0.08, − 0.03)*	*− 0.06 (− 0.09, − 0.03)*	686	33.0%	*1.25 (1.16, 1.35)*	1.19 (1.05, 1.34)*
Pneumonia	− 0.25	0.53	− 0.01 (− 0.06, 0.04)	−0.0002 (− 0.05, 0.05)	120	28.6%	1.00 (0.85, 1.17)	0.92 (0.76, 1.10)*
Malaria	− 0.29	0.53	*− 0.06 (− 0.09, − 0.03)*	− 0.03 (− 0.06, 0.01)	515	34.1%	*1.27 (1.16, 1.38)*	1.15 (1.01, 1.31)*
Malnutrition	−0.31	0.47	*− 0.07 (− 0.12, − 0.01)*	*− 0.19 (− 0.24, − 0.13)*	116	33.2%	*1.17 (1.01, 1.37)*	1.52 (1.25, 1.85)*
Other bacterial infection	− 0.39	0.57	*− 0.15 (− 0.26, − 0.04)*	− 0.08 (− 0.19, 0.02)	40	48.8%	*1.72 (1.37, 2.15)*	1.32 (0.96, 1.83)*
Upper respiratory tract infection	− 0.25	0.69	− 0.0004 (− 0.34, 0.34)	0.07 (− 0.27, 0.41)	1	14.3%	0.59 (0.103.63)	0.54 (008, 3.83)*
IMCI danger signs								
None	− 0.10	0.49	Reference	Reference	580	22.8%	Reference	Reference
At least 1	− 0.28	0.51	*− 0.07 (− 0.09, − 0.04)*	*− 0.09 (− 0.11, − 0.06)*	1196	32.7%	*1.43 (1.31, 1.56)*	1.35 (1.22, 1.50)*
3 signs present	− 0.27	0.58	− 0.08 (− 0.20, 0.0.04)	− 0.05 (− 0.17, 0.07)	21	31.8%	1.39 (0.90, 2.16)	1.29 (0.83, 2.00)*
2 signs present	− 0.29	0.49	*− 0.10 (− 0.14, − 0.07)*	*− 0.07 (− 0.11, − 0.04)*	396	34.1%	*1.49 (1.32, 1.70)*	1.32 (1.15, 1.51)*
1 sign present	− 0.28	0.49	**−***0.08 (− 0.11, − 0.05)*	*− 0.07 (− 0.10, − 0.04)*	777	32.0%	*1.40 (1.26, 1.56)*	1.37 (1.22, 1.53)*
Access to improved water								
No	− 0.25	0.51	Reference	Reference	1007	29.8%	Reference	Reference
Yes	− 0.24	0.49	0.01 (− 0.01, 0.04)	*− 0.04 (− 0.07, − 0.01)*	769	27.2%	0.91 (0.83, 1.00)	1.09 (0.96, 1.23)*
Improved defecation facility								
No	− 0.26	0.50	Reference	Reference	1464	29.0%	Reference	Reference
Yes	− 0.21	0.49	*0.05 (0.01, 0.08)*	*0.07 (0.03, 0.11)*	312	27.1%	0.93 (0.83, 1.05)	0.87 (0.74, 1.01)*
Wealth index								
Lowest quintile	− 0.25	0.49	Reference	Reference	344	28.3%	Reference	Reference
Second lowest	− 0.28	0.52	− 0.03 (− 0.07, 0.01)	− 0.01 (− 0.05, 0.03)	399	32.0%	*1.13 (1.00, 1.28)*	1.06 (0.91, 1.22)*
Middle	− 0.25	0.50	0.005 (− 0.03, 0.04)	0.03 (− 0.01, 0.07)	387	28.8%	1.02 (0.90, 1.15)	0.95 (0.82, 1.10)*
Second highest	− 0.23	0.47	0.03 (− 0.01, 0.07)	0.04 (− 0.001, 0.08)	296	25.5%	0.90 (0.79, 1.03)	0.92 (0.79, 1.08)*
Highest quintile	− 0.21	0.52	0.03 (− 0.07, 0.01)	*0.08 (0.04, 0.12)*	347	28.2%	0.99 (0.86, 1.13)	0.90 (0.77, 1.04)*

^a^Analyses of age and stunting were not adjusted for age and baseline LAZ, respectively

^b^Analysis of age as a risk factor was not adjusted for age

^c^Analysis of stunting was not adjusted for baseline LAZ

^d^Data on duration of diarrhea for the 7 days before enrollment were ascertained at enrollment (children with diarrhea lasting longer than 7 days were excluded at this point), and data on diarrhea duration for the 14 days following enrollment were ascertained with a memory aid suitable for groups of all literacy levels, which the caregiver returned at the 60-day follow-up visit

Several clinical factors at MSD presentation were associated with ΔLAZ. Children who were hospitalized at enrollment lost 0.11 more LAZ than those who were not (95% CI − 0.14, − 0.07) and those who presented with fever lost 0.09 more LAZ (95% CI − 0.09, − 0.06) in adjusted analysis. Children presenting with at least one Integrated Management of Childhood Illness (IMCI) danger sign lost more LAZ than those who had none (aβ − 0.05 [95% CI − 0.08, − 0.02]). Presentation with any co-morbidity was associated with losing more LAZ (aβ − 0.09 [95% CI − 0.11, − 0.06]), but this association was likely driven by one specific co-morbidity: among the co-morbidities documented in medical records, only a discharge diagnosis of malnutrition was associated with loss of LAZ in the adjusted analysis. Compared to children with non-dysenteric MSD, those presenting with dysentery lost less LAZ (aβ 0.07 [95% CI 0.07, 0.11]). Prolonged or persistent MSD (using caregiver-recalled duration of diarrhea at follow-up) was also not associated with linear growth faltering.

In addition to clinical factors, several baseline socio-demographic factors were also protective against loss of LAZ. Children whose caregivers reported access to an improved defecation facility lost substantially less LAZ than those without access to this level of sanitation (aβ 0.07 [95% CI 0.03, 0.11]) though access to improved water sources were not significantly associated. In addition, children in the highest wealth quintile lost less LAZ than those in the lowest quintile (aβ 0.08 [95% CI 0.04, 0.12]).

Using multiple imputation resulted in an additional 854 children being added to the dataset, resulting in 7057 included in the analysis with imputed outcomes. An additional file presents distribution of imputed versus observed outcomes (Additional file 1: Figure S1), as well as baseline characteristics between children with imputed versus observed outcomes (Additional file 1: Table S1). Factors associated with ΔLAZ calculated using imputed values were similar to the complete-case analysis (Additional file 1: Table S2), with no substantial differences in effect size or statistical significance.

#### Severe linear growth faltering (loss ≥ 0.5 LAZ)

**Fig. 3:**
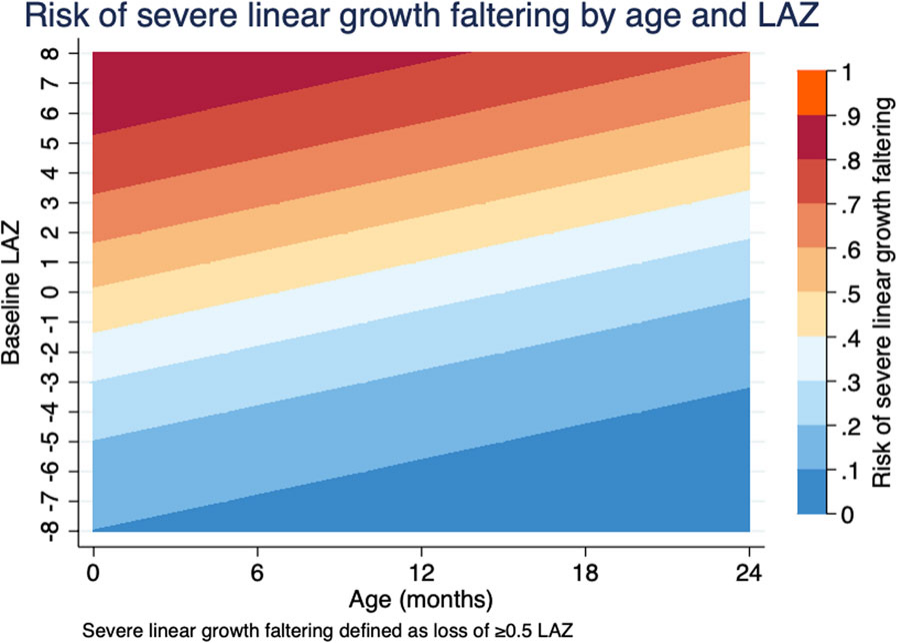
Risk of linear growth faltering in terms of interactions between age and baseline LAZ

Prevalence of severe linear growth faltering by age and nutritional status at presentation followed a similar pattern to that of ΔLAZ (Table 2 and Fig. 2c/d); children 0–6 months of age were more likely to experience severe linear growth faltering than children > 12–23 months (aPR 1.41 [95% CI 1.22, 1.51]). We also depict the pattern of prevalence of severe linear growth faltering by interactions between age and baseline LAZ (Fig. 3). Unlike our results for ΔLAZ, female children were 9% less likely to experience severe linear growth faltering than males (aPR 0.91 [95% CI 0.83, 1.00]). Hospitalization, fever, and at least one IMCI danger sign were significant risk factors for severe linear growth faltering, as they were for ΔLAZ. Non-dysenteric MSD did not emerge as a statistically significant risk factor for severe linear growth faltering (aPR 0.88 [95% CI 0.75, 1.02]), but the prevalence ratio did approach statistical significance (*p* value = 0.09). Unlike our results for ΔLAZ, the socio-demographic factors examined (improved water source or defecation facility, and wealth quintile) were not statistically significantly associated with severe linear growth faltering in our analyses.

Results for the analysis including imputed values were similar (Additional file 1: Table S2).

### Prediction model results

In the derivation dataset of 3101 children, there were 894 who experienced severe linear growth faltering (28.8%). The validation cohort also consisted of 3102 children, of whom 882 (28.4%) experienced severe linear growth faltering. Demographic and clinical characteristics were similar between the derivation and validation datasets (Table 3).

**Table 3:** Select characteristics of children in the derivation and validation datasets

	Derivation *N* = 3101		Validation *N* = 3102	
	*n* (%) or median (interquartile range)		*n* (%) or median (interquartile range)	
Sociodemographic characteristics				
Age, months	11	(7–16)	11	(7–16)
0–6 months	559	(18.0%)	518	(16.7%)
> 6–12 months	1192	(38.4%)	1169	(37.7%)
> 12–23 months	1350	(43.5%)	1415	(45.6%)
Site				
The Gambia	346	(11.2%)	359	(11.6%)
Mali	609	(19.6%)	563	(18.1%)
Mozambique	193	(6.2%)	217	(7.0%)
Kenya	476	(15.3%)	485	(15.6%)
India	625	(20.2%)	570	(18.4%)
Bangladesh	493	(15.9%)	500	(16.1%)
Pakistan	359	(11.6%)	408	(13.2%)
Female	1358	(43.8%)	1323	(42.6%)
Access to improved water	1420	(45.8%)	1404	(45.3%)
Access to improved sanitation^a^	559	(18.0%)	594	(19.1%)
Wealth index [13]	− 0.08	(− 0.71, 0.59)	− 0.08	(− 0.72, 0.58)
Clinical characteristics at presentation				
Stunting	715	(23.1%)	763	(24.6%)
Wasting	671	(21.6%)	686	(22.1%)
Severe acute malnutrition	227	(7.3%)	243	(7.9%)
MUAC < 12.5 cm among 6–23 months	421	(16.6% of 2542)	442	(17.2% of 2542)
Fever	886	(30.0%)	902	(30.5%)
Current breastfeeding < 6 months				
Exclusive	220	(29.4% of 749)	219	(31.0% of 707)
Partial	498	(66.5% of 749)	453	(64.1% of 707)
None	31	(4.1% of 749)	35	(5.0% of 707)
Hospitalized at presentation	605	(19.5%)	624	(20.1%)
Dysentery at presentation^b^	681	(22.0%)	671	(21.6%)
≥ 1 IMCI general danger sign	1808	(58.3%)	1853	(59.7%)
Presented with at least 1 co-morbidity^c^	1027	(33.1%)	1049	(33.8%)
Pneumonia	2880	(92.9%)	2903	(93.6%)
Malaria	221	(7.1%)	199	(6.4%)
Malnutrition	741	(23.9%)	769	(24.8%)
Other invasive bacterial infection	161	(5.2%)	188	(6.1%)
Upper respiratory tract infection	47	(1.5%)	35	(1.1%)

^a^Flush toilet, ventilated improved pit latrine with or without water seal, or pour flush toilet not shared with other households

^b^Visible blood in stool observed by study staff or reported by caregiver at presentation; discharge diagnosis of dysentery per managing clinician upon leaving the healthcare facility; or observed in stool sample by laboratory staff

^c^Per discharge diagnoses documented on medical records

**Fig. 4:**
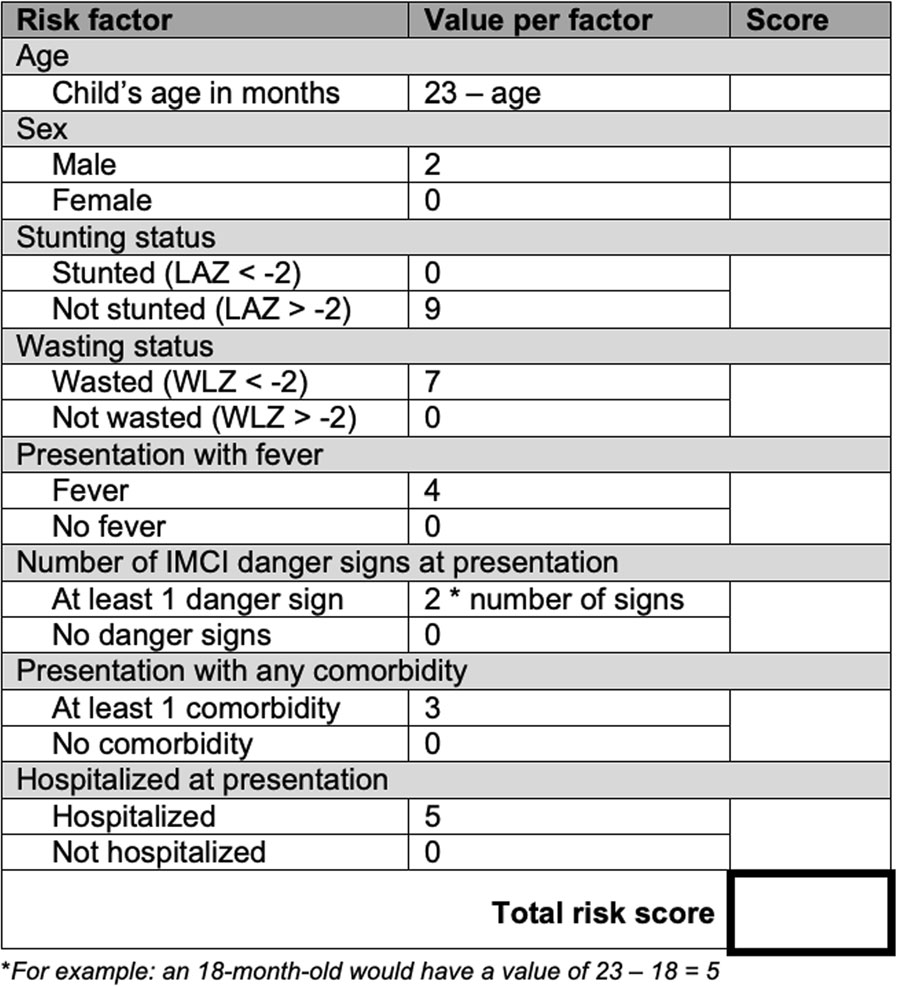
A risk scoring tool for predicting risk of linear growth faltering among children presenting with MSD

**Fig. 5:**
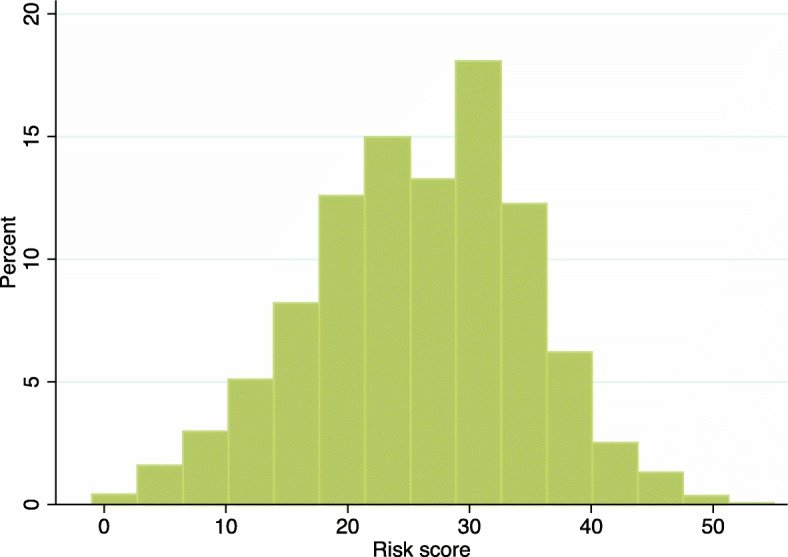
Distribution of risk scores among all children with complete outcome data (n = 6203)

**Fig. 6:**
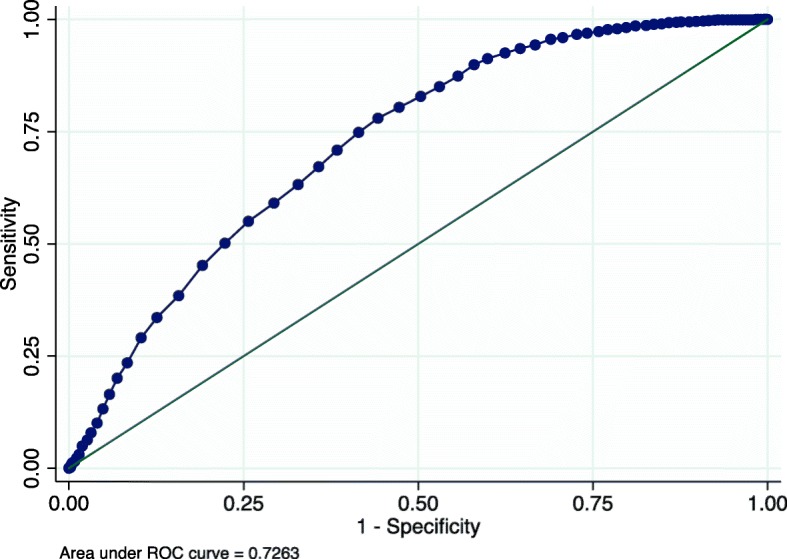
ROC curve of predicted risks of severe linear growth faltering using risk scores in the derivation cohort

The final prediction model included age, sex, stunting at enrollment, wasting at enrollment, presentation with fever, presentation with at least 1 IMCI danger sign, presentation with any comorbidity, and admission to hospital at enrollment. These factors were used to create a risk score for severe linear growth faltering each child (Fig. 4). In the overall cohort, risk scores ranged from 0 to 55, and the median risk score was 27 (interquartile range 20–32) (Fig. 5). Mean variance inflation factor was 1.9. Model fit was similar in the derivation and validation datasets (AUC 0.73 (95% CI 0.71, 0.74); 0.73 (95% CI 0.72, 0.74), respectively) (Fig. 6). In the derivation dataset, a cutoff of 27 optimized both sensitivity and specificity at 63.2% and 67.2%, respectively (Table 4). In the validation dataset, the sensitivity, specificity, PPV, and NPV of the cutoff point of 27 in the validation dataset were 60.6%, 69.9%, 44.7%, and 81.6%, respectively. Also in the validation dataset, the risk score identified children most likely to severely growth falter better than any individual predictive factor: age (AUC = 0.31 [95% CI 0.30, 0.33]), sex (AUC = 0.49 [95% CI 0.47, 0.50]), stunting (AUC = 0.44 [95% CI 0.43, 0.45]), wasting (AUC = 0.53 [95% CI 0.52, 0.54]), presentation with fever (AUC = 0.55 [95% CI 0.53, 0.56]), presentation with at least 1 IMCI danger sign (AUC = 0.57 [95% CI 0.55, 0.58]), presentation with any comorbidity (AUC = 0.53 [95% CI 0.47, 0.50]), and hospitalization at presentation (AUC = 0.55 [95% CI 0.54, 0.56]).

**Table 4:** Sensitivity, specificity, and predictive values of risk score at different cut-points in the derivation cohort

	Risk score cut-point				
	≥ 11	≥ 20	≥ 27	≥ 32	≥ 40
No. of children (% of total [6203])	5654 (91.2%)	4567 (73.4%)	3015 (48.6%)	1632 (26.3%)	324 (5.2%)
Sensitivity	96.7%	85.0%	63.2%	38.5%	7.9%
Specificity	27.3%	47.0%	67.2%	84.3%	96.8%
Positive predictive value	34.6%	38.9%	43.3%	49.3%	49.9%
Negative predictive value	90.6%	86.6%	81.2%	77.0%	72.4%

## Discussion

In this post hoc analysis of children with MSD enrolled in the GEMS study, we found that over one-fifth of children under 24 months had linear growth faltering at ~ 60-days following the MSD episode. We identified several risk factors for linear growth faltering, including age, fever, general IMCI danger sign, and nutritional status. We found that some of these factors yielded reasonable predictive value to identify children likely to experience severe linear growth faltering following MSD. We found that most children who experienced linear growth faltering were not stunted at MSD presentation. Stunting status at diarrhea presentation may not identify all children who are at risk for linear growth declines following an episode of MSD. Using these other clinical factors to predict linear growth faltering may result in earlier and more complete identification of children who are on a trajectory of linear growth declines, comparing to using only stunting status at diarrhea presentation to predict post-diarrhea growth declines and thus may be useful for targeting interventions to prevent stunting.

Patterns of linear growth faltering followed patterns determined by age and baseline LAZ. The older the child, or the lower the LAZ value at enrollment, the lower the probability that the child with lose LAZ over the ensuing 2–3 months. Growth in early life is rapid and decreases as the child ages [20, 21]. Correspondingly, risks of linear growth faltering decrease as children age, with the highest risk occurring before 12 months. Our findings are consistent with previous work noting the substantial losses of LAZ in early life [9] and suggest that interventions may confer the most benefit within this critical period. This growth pattern also underscores methodological considerations for analyses of linear growth faltering. Children in the youngest age groups have the highest growth velocity and therefore have the greatest opportunity to lose or gain LAZ. Growth faltering in older children may have different underlying etiologies and health implications than that occurring in infancy, and so we have restricted the analysis to children under 24 months of age and controlled for age and baseline LAZ in the analysis. Challenges in ascertaining and interpreting losses in linear growth by age group highlight the need for research to assess the clinical relevance of different magnitudes of loss in LAZ by different age groups.

Similar to the patterns of LAZ loss by age, children with LAZ values that are already low (below 0) were less likely to lose more LAZ. We have described how losses in LAZ increase consistently with higher LAZ, consistent with patterns observed elsewhere [22]. While our results suggest stunting status or low LAZ values may not identify children who are at risk for *further* linear growth deterioration, children who are already stunted are at high risk of the health and cognitive detriments associated with chronic malnutrition [5]. Whether health consequences of loss in LAZ are differential by stunting status remain unclear. A modest loss in LAZ may prove to have more health consequences in already stunted children than a loss of greater magnitude in non-stunted children at diarrhea presentation.

We identified host, clinical, and environmental characteristics that were significantly associated with short-term linear growth faltering. Acute malnutrition (measured either by MUAC or WLZ) was significantly associated with subsequent growth faltering. This could also reflect dehydration status, and this finding may reflect that the more dehydrated children had more severe intestinal injury and absorptive capacity, and thus more likely to experience linear growth faltering. Ponderal growth has previously been found to be associated with linear growth. A longitudinal analysis of birth cohorts from the USA, Ghana, and Honduras reported that WLZ was positively correlated with length gain [23], as did a cohort study of Jamaican 9–24-month-old stunted children [24]. Additionally, a study in the West Indies reported that severely malnourished children needed to attain ≥ 85% WLZ before they began to gain LAZ [25]. These studies suggest ponderal growth may precede linear growth, as weight loss reflects a lack of available nutrients needed to sustain linear growth. It is also possible the higher risks of severe linear growth faltering we observed in acutely malnourished children may be due to higher rates of subsequent diarrhea episodes during the follow-up period. Previous research has reported higher incidence of diarrhea in acutely malnourished children [26, 27], though we did not have data on diarrhea beyond 14 days of follow-up to examine this hypothesis. Acutely malnourished children presenting with MSD may thus be an easily identifiable population who may benefit from nutritional interventions that protect against linear growth faltering.

Presentation with fever was associated with linear growth faltering as has been shown previously [28]. Fever may be a sign of more severe intestinal inflammation and injury, as is often seen in bacterial diarrhea, which may be associated with linear growth faltering. This is supported by the finding that children with MSD who were hospitalized at presentation were at higher risk of linear growth faltering than those who were not. Finally, the presence of any IMCI danger sign at MSD presentation was also associated with a loss of more LAZ. Studies have demonstrated the potential of IMCI programs for improving quality of care and child survival [29–31]. However, a Cochrane review of the effectiveness of IMCI programs reported little to no benefit on stunting or wasting [30] which could reflect the lack of effective interventions for improving nutritional status upon identification of high-risk children.

In our analysis, children presenting with dysentery had lower risks of linear growth faltering than those with non-dysenteric MSD. This finding was unexpected and differs from that of other studies that found dysentery, or specific pathogens known to cause dysentery, to be associated with risk for linear growth faltering [32–34]. Our detection of a *reduced* risk associated with dysentery may be related to clinical management. WHO guidelines recommend antibiotics for dysentery [35], and in our data, children presenting with dysentery were more likely to receive an antibiotic than those without. It is unclear whether antibiotic management of MSD alters growth [36]; some research has reported growth-promoting effects of antibiotic treatment on length and weight in children in LMICs [37, 38]. Clinical trial data will be needed for evaluating the effectiveness of antibiotic management of MSD for protecting against subsequent linear growth faltering.

We found that children in lower wealth quintiles had the highest rates of linear growth faltering. Poverty is a well-established underlying cause of childhood stunting. There are large disparities in stunting rates by wealth quintile within LMICs, with child stunting rates in lowest wealth quintiles as much as 13 times higher than in the highest [39]. Socioeconomic factors are the most consistently identified correlates of stunting [40], and it has been estimated that every 10% increase in national gross domestic production per person would result in a 6% decrease in stunting prevalence [41]. Economic development may be influential in protecting children with MSD against linear growth faltering [42]. We found that children in households without access to improved defecation facilities experienced greater losses in LAZ, though results were not consistent for both outcomes and for our analyses of water source. Greater exposure to environmental pathogens may place children at higher risk of linear growth faltering, as pathogen-specific diarrhea [33, 34] and asymptomatic pathogen carriage [43–45] have been found to be associated with linear growth faltering. Unimproved WASH may also contribute to environmental enteric dysfunction (EED), which is strongly associated with linear growth faltering and thought to play a central role in stunting [46, 47]. However, WASH interventions have not yielded consistent benefits. While a review of stunting in 137 LMICs using Global Burden of Disease data reported unimproved sanitation to be a leading cause of stunting [6], a Cochrane review reported only modest benefits of WASH on child length but limited availability and quality of evidence [48]. Large clinical trials of WASH interventions did not detect a benefit on child growth [49–51].

When considering which risk factors best predicted likelihood of severe linear growth faltering, age, stunting, wasting, fever, and presence of any IMCI danger sign emerged as the most important. The predictive ability of the model was fair to moderate and could benefit from further research to improve the predictive ability of the model, perhaps by including data not available in GEMS, such as birth weight or HIV status information. External validation would further improve the model. The risk score model performed better than any individual predictive factor, suggesting that the combination of these factors is more useful for identifying children at risk of severe linear growth faltering than any of these variables individually. We identified the risk score cut-point that maximizes sensitivity and specificity, but the cut-point used in practice should be weighed against the costs or negative consequences of potential interventions. This predictive model uses only easily collected clinical data routinely documented at diarrhea presentation, and such a risk score could be useful for identifying children at highest risk for inclusion in trials of interventions to reduce linear growth faltering and ultimately may prove useful in determining how to best apply successful interventions once benefit is demonstrated, by identifying high-risk children who stand to benefit from such an intervention or be monitored more closely following MSD.

There have been few studies to our knowledge that identify risk factors of linear growth faltering in children following an episode of MSD. Our study contributes data on this important topic, using a large, multi-country cohort with a rigorous study design and data collection practices. There are several limitations to our analysis as well. Data on birth size, HIV status, and previous and subsequent diarrhea episodes were not available in the parent study, which may be relevant to this secondary analysis. Our analysis assessed short-term effects (2–3 months) only. It has been reported that catch-up growth is possible following a diarrhea episode if no subsequent diarrhea episodes are experienced [52], and it is possible that some of the growth deficits we observed were transient. The risk and predictive factors we have identified for short-term losses in LAZ may or may not be the same factors associated with longer term growth declines. However, we found that a substantial proportion of these children presenting with MSD experienced a repeated diarrhea episode in the subsequent 50–90 days, and this additional growth insult may have precluded catch-up growth for this subset, who may have continued on a linear growth decline. This short-term period also represents a very vulnerable period, as children’s risk of death was more than 8-fold in this period following a MSD episode compared to healthy controls [8]. Longer follow-up studies will be important for assessing sustained linear growth deficits associated with diarrhea, as well as other health outcomes associated with linear growth faltering at different ages. The cut-off of 0.5 LAZ for our definition of severe linear growth faltering is arbitrary, and the clinical implications of this magnitude of loss are unclear. Additionally, all definitions used implicitly assume the impact of LAZ loss is the same, irrespective of age or enrollment LAZ. We adjusted for age and LAZ at baseline in our analysis, but difficulties remain with interpreting the health detriments of these outcomes.

## Conclusion

Children presenting with MSD that are acutely malnourished (or more dehydrated), under 12 months of age, presenting with more severe disease (as indicated by hospitalization, presence of fever, or IMCI danger signs), and those living with limited access to improved sanitation may be at higher risk of linear growth faltering following MSD. To identify children for inclusion in further trials and to guide clinical decision-making for close monitoring of high-risk children or targeting an intervention once an effective intervention has been identified, age, nutritional status, and signs of disease severity may be useful to identify children at highest risk.

## Supplementary information

**Additional file 1: Figure S1.** Distribution of observed versus imputed outcome values. **Table S1.** Enrollment characteristics of GEMS cases included in the present analysis of growth faltering. **Table S2.** Risk factors for linear growth faltering among children 0–23 months old with MSD including imputed outcome data.

## Data Availability

The data are available in the GEMS Repository: https://clinepidb.org/ce/app/.

## Abbreviations

95% CI: 95% confidence interval
AIC: Akaike Information Criterion
AUC: Area under the curve
GEMS: Global Enteric Multicenter Study
HAZ/LAZ: Height/length-for-age z-score
LMIC: Low- and middle-income country
MSD: Moderate-to-severe diarrhea
MUAC: Middle-upper arm circumference
NPV: Negative predictive value
PPV: Positive predictive value
PR: Prevalence ratio
ROC: Receiver operating characteristic
SD: Standard deviation
WHO: World Health Organization
