# Supplementary material for: Determinants of linear growth faltering among children with moderate-to-severe diarrhea in the Global Enteric Multicenter Study
Source: BMC Med. 2019 Nov 25;17:214. doi: 10.1186/s12916-019-1441-3 (PMC6878715; doi:10.1186/s12916-019-1441-3)
Supplement: Supplementary file 1 — Additional file 1: Figure S1. Distribution of observed versus imputed outcome values. Table S1. Enrollment characteristics of GEMS cases included in the present analysis of growth faltering. Table S2. Risk factors for linear growth faltering among children 0–23 months old with MSD including imputed outcome data. [file 12916_2019_1441_MOESM1_ESM.docx]

Additional file 1: Table S1. Enrollment characteristics of GEMS cases included in the present analysis of growth faltering

|  | | Children with complete data  N=6,203 | | Children with imputed outcomes  N=854 | | p-value for difference |
| --- | --- | --- | --- | --- | --- | --- |
|  | | n(%) or median (interquartile range) | | n(%) or median (interquartile range) | | t-test or chi2 |
| Sociodemographic characteristics | | | |  |  |  |
| Age, months | | 11 | (7-16) | 10 | (7-15) | **P=0.0051** |
|  | 0-6 months | 1077 | (17.4%) | 156 | (18.3%) | **P=0.015** |
|  | >6-12 months | 2361 | (38.1%) | 351 | (41.2%) |  |
|  | >12-23 months | 2765 | (44.6%) | 344 | (40.4%) |  |
| Site | |  |  |  |  | **P<0.0001** |
|  | the Gambia | 705 | (11.4%) | 125 | (14.7%) |  |
|  | Mali | 1172 | (18.9%) | 207 | (24.3%) |  |
|  | Mozambique | 410 | (6.6%) | 110 | (12.9%) |  |
|  | Kenya | 961 | (15.5%) | 87 | (10.2%) |  |
|  | India | 1195 | (19.3%) | 54 | (6.3%) |  |
|  | Bangladesh | 993 | (16.0%) | 21 | (2.5%) |  |
|  | Pakistan | 767 | (12.4%) | 247 | (29.0%) |  |
| Female | | 2681 | (43.2%) | 388 | (45.6%) | P=0.157 |
| Access to improved water | | 2824 | (45.5%) | 283 | (33.3%) | **P<0.0001** |
| Access to improved sanitation^[[1]](#endnote-1)^ | | 1153 | (18.6%) | 183 | (21.5%) | **P=0.028** |
| Wealth quintile^14^ | | -0.08 | (-0.71, 0.59) | -0.26 | (-0.87, 0.36) | **P<0.0001** |
| Clinical characteristics at presentation | | | |  |  |  |
| Stunting | | 1478 | (23.8%) | 292 | (34.5%) | **P<0.0001** |
| Wasting | | 1357 | (21.9%) | 283 | (33.5%) | **P<0.0001** |
| Severe acute malnutrition | | 470 | (7.6%) | 130 | (15.4%) | **P<0.0001** |
| MUAC < 12.5 cm among 6-23 mos | | 863 | (16.8% of 5126 children) | 176 | (28.0% of 640) | **P<0.0001** |
| Fever | | 1788 | (30.3%) | 287 | (35.1%) | **P=0.007** |
| Current breastfeeding < 6 mos | |  |  |  |  | **P<0.0001** |
|  | Exclusive | 439 | (30.2%) | 77 | (36.5%) |  |
|  | Partial | 951 | (65.3%) | 118 | (55.9%) |  |
|  | None | 66 | (4.5%) | 16 | (7.6%) |  |
| Hospitalized at presentation | | 1141 | (19.8%) | 187 | (22.0%) | **P=0.0214** |
| Dysentery at presentation^[[2]](#endnote-2)^ | | 1375 | (21.8%) | 99 | (11.6%) |  |
| ≥1 IMCI general danger sign | | 3426 | (59.0%) | 594 | (69.8%) | **P<0.0001** |
| Presented with at least 1 co-morbidity^[[3]](#endnote-3)^ | | 1911 | (33.5%) | 364 | (42.8%) | **P<0.0001** |
|  | Pneumonia | 420 | (6.8%) | 83 | (9.8%) | **P=0.002** |
|  | Malaria | 1510 | (24.3%) | 219 | (25.7%) | P=0.428 |
|  | Malnutrition | 349 | (5.6%) | 110 | (12.9%) | **P<0.0001** |
|  | Other invasive bacterial infection | 82 | (1.3%) | 18 | (2.1%) | P=0.06 |
|  | Upper respiratory tract infection | 8 | (0.1%) | 0 | (0%) | P=0.273 |

Additional file 1: Figure S1. Distribution of observed vs imputed outcome values

1. Change in LAZ b. Prevalence of severe linear growth faltering (loss of ≥1 LAZ)

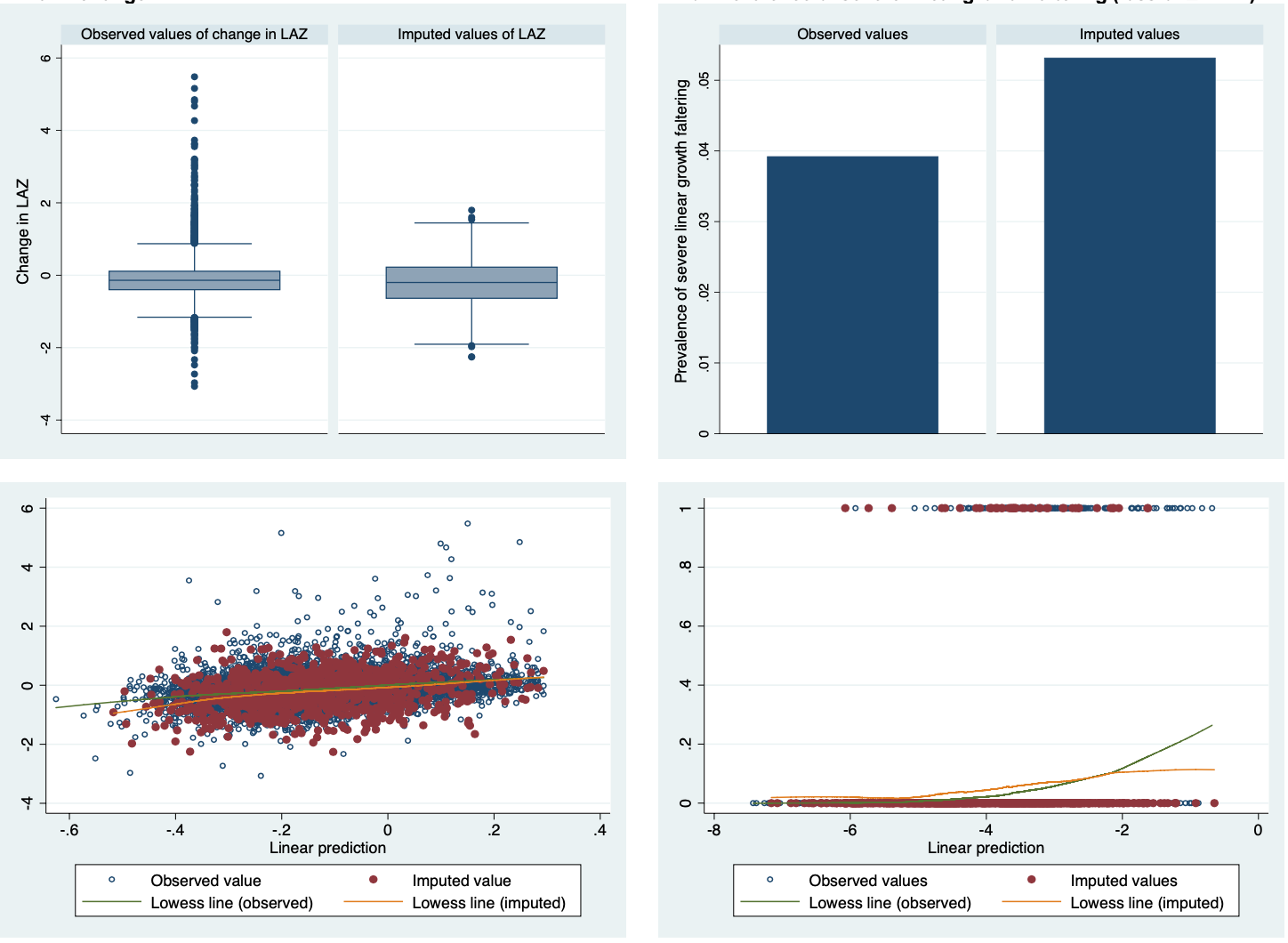

Additional file 1: Table S2. Risk factors for linear growth faltering among children 0-23 months old with MSD including imputed outcome data. Statistically significant results (p <0.05) are bolded.

|  | | Severe linear growth faltering | | | | | Change in LAZ | | | | | |
| --- | --- | --- | --- | --- | --- | --- | --- | --- | --- | --- | --- | --- |
|  | | **n^[[4]](#endnote-4)^** | **%** | **Crude relative risks** | | **Adjusted for age, site, and baseline LAZ** | | **mean** | **SD** | | **Crude difference in change in LAZ** | **Adjusted for age, site, and baseline LAZ** |
| *Age* | |  |  |  |  | |  | |  | | | |
|  | 0-6 mo | 431 | 35.0% | **1.58 (1.42, 1.74)** | **1.42 (1.20, 1.47)** | | -0.27 | | 0.70 | **-0.08 (-0.11, -0.04)** | | **-0.02 (-0.10, -0.05)** |
|  | >6-12 mo | 875 | 32.2% | **1.45 (1.33, 1.58)** | **1.33 (1.25, 1.61)** | | -0.31 | | 0.49 | **-0.12 (-0.14, -0.09)** | | **-0.07 (-0.06, -0.01)** |
|  | >12-23 mo | 690 | 22.2% | Reference | Reference | | -0.19 | | 0.40 | Reference | | Reference |
| Sex | |  |  |  |  | |  | |  |  | |  |
|  | Male | 1233 | 23.7% | Reference | Reference | | -0.25 | | 0.53 | Reference | | Reference |
|  | Female | 892 | 22.3% | 0.95 (0.88, 1.02) | 0.94 (0.86, 1.02) | | -0.24 | | 0.47 | 0.01 (-0.01, 0.04) | | 0.02 (-0.0003, 0.05) |
| Stunting | |  |  |  |  | |  | |  |  | |  |
|  | No | 1612 | 30.5% | Reference | Reference | | -0.29 | | 0.49 | Reference | | Reference |
|  | Yes | 377 | 21.3% | **0.70 (0.63, 0.77)** | **0.72 (0.65, 0.81)** | | -0.12 | | 0.50 | **0.17 (0.14, 0.20)** | | **0.16 (0.13, 0.19)** |
| Wasting | |  |  |  |  | |  | |  |  | |  |
|  | No | 1432 | 26.5% | Reference | Reference | | -0.22 | | 0.51 | Reference | | Reference |
|  | Yes | 554 | 33.8% | **1.27 (1.17, 1.38)** | **1.52 (1.37, 1.68)** | | -0.34 | | 0.47 | **-0.12 (-0.15, -0.09)** | | **-0.21 (-0.24, -0.18)** |
| MUAC (among >6 mos) | |  |  |  |  | |  | |  |  | |  |
|  | ≥12.5 | 1236 | 25.9% | Reference | Reference | | -0.24 | | 0.45 | Reference | | Reference |
|  | <12.5 cm | 329 | 31.1% | 1.20 (1.06, 1.35) | **1.39 (1.22, 1.60)** | | -0.27 | | 0.46 | **-0.04 (-0.07, -0.004)** | | **-0.12 (-0.15, -0.08)** |
| Current breastfeeding (among < 6 mos) | |  |  |  |  | |  | |  |  | |  |
|  | Exclusive | 171 | 33.1% | Reference | Reference | | -0.30 | | 0.73 | Reference | | Reference |
|  | Partial | 356 | 33.2% | 1.00 (0.86, 1.16) | 1.06 (0.87, 1.31) | | -0.25 | | 0.68 | 0.04 (-0.04, 0.14) | | -0.01 (-0.10, 0.09) |
|  | None | 28 | 34.2% | 1.03 (0.74, 1.43) | 1.20 (0.80, 1.82) | | -0.28 | | 0.52 | 0.01 (-0.20, 0.23) | | -0.11 (-0.32, 0.11) |
| Diarrhea type^[[5]](#endnote-5)^ | |  |  |  |  | |  | |  |  | |  |
|  | Acute | 841 | 26.6% | Reference | Reference | | -0.23 | | 0.48 | Reference | | Reference |
|  | Prolonged | 579 | 29.8% | 1.12 (1.01, 1.25) | 1.09 (0.98, 1.21 | | -0.26 | | 0.50 | **-0.03 (-0.06, -0.003)** | | -0.01 (-0.04, 0.01) |
|  | Persistent | 204 | 30.0% | 1.13 (0.97, 1.32) | 1.12 (0.96, 1.31) | | -0.26 | | 0.50 | -0.03 (-0.07, 0.01) | | -0.003 (-0.04, 0.04) |
| Hospitalized at enrollment | |  |  |  |  | |  | |  |  | |  |
|  | No | 1572 | 21.2% | Reference | Reference | | -0.23 | | 0.49 | Reference | | Reference |
|  | Yes | 553 | 31.3% | **1.35 (1.24, 1.47)** | **1.35 (1.20, 1.52)** | | -0.32 | | 0.54 | -0.09 (-0.12, -0.06) | | **-0.11 (-0.14, -0.07)** |
| Presentation with fever | |  |  |  |  | |  | |  |  | |  |
|  | No | 1194 | 25.7% | Reference | Reference | | -0.21 | | 0.51 | Reference | | Reference |
|  | Yes | 683 | 32.9% | **1.28 (1.19, 1.39)** | **1.20 (1.09, 1.33)** | | -0.31 | | 0.49 | **-0.10 (-0.13, -0.07)** | | **-0.09 (-0.12, -0.06)** |
| Presentation with dysentery | |  |  |  |  | |  | |  |  | |  |
|  | No | 1630 | 29.1% | Reference | Reference | | -0.20 | | 0.49 | Reference | | Reference |
|  | Yes | 360 | 25.2% | 0.87 (0.79, 0.96) | 0.82 (0.71, 0.94) | | -0.26 | | 0.51 | **0.06 (0.03, 0.079** | | **0.08 (0.04, 0.11)** |
| Co-morbidities | |  |  |  |  | |  | |  |  | |  |
|  | None | 1199 | 26.0% | Reference | *Reference* | | -0.23 | | 0.50 | Reference | | Reference |
|  | Any | 797 | 32.6% | **1.26 (1.17, 1.35)** | **1.22 (1.08, 1.37)** | | -0.28 | | 0.51 | **-0.06 (-0.08, -0.03)** | | **-0.06 (-0.09, -0.03)** |
|  | Pneumonia | 143 | 28.4% | 1.00 (0.87, 1.16) | 0.93 (0.78, 1.10)) | | -0.25 | | 0.53 | -0.01 (-0.06, 0.04) | | -0.0002 (-0.05, 0.05) |
|  | Malaria | 577 | 33.4% | **1.25 (1.16, 1.36)** | **1.15 (1.02, 1.30)** | | -0.29 | | 0.51 | **-0.06 (-0.09, -0.03** | | -0.03 (-0.07, 0.01) |
|  | Malnutrition | 156 | 33.9% | **1.22 (1.06, 1.39)** | **1.50 (1.26, 1.78)** | | -0.31 | | 0.47 | **-0.07 (-0.12, -0.01)** | | **-0.19 (-0.24, -0.13)** |
|  | Other bacterial infection | 49 | 49.0% | **1.75 (1.42, 2.14)** | **1.38 (1.03, 1.85*)*** | | -0.39 | | 0.57 | -**0.15 (-0.26, -0.04)** | | -0.08 (-0.19, 0.02) |
|  | Upper respiratory tract infection | 2 | 25.0% | 0.88 (0.27, 2.93) | 0.72 (0.18, 2.90) | | -0.25 | | 0.69 | -0.0005 (-0.35, 0.35) | | 0.07 (-0.27, 0.41) |
| IMCI danger signs | |  |  |  |  | |  | |  |  | |  |
|  | None | 639 | 22.8% | Reference | Reference | | -0.20 | | 0.49 | Reference | | *Reference* |
|  | At least 1 | 1357 | 31.9% | **1.33 (1.18, 1.50)** | **1.33 (1.20, 1.47)** | | -0.28 | | 0.51 | **-0.09 (-0.11, -0.06)** | | **-0.07 (-0.09, -0.04)** |
|  | 3 signs present | 26 | 29.2% | 1.28 (0.86, 1.89) | 1.12 (0.74, 1.68) | | -0.27 | | 0.58 | **-0.08 (-0.11, -0.05)** | | *-*0.05 (-0.17, 0.07) |
|  | 2 signs present | 452 | 33.4% | **1.46 (1.29, 1.65)** | **1.31 (1.15, 1.49)** | | -0.30 | | 0.53 | **-0.10 (-0.14, -0.07)** | | **-0.07 (-0.11, -0.04)** |
|  | 1 sign present | 877 | 31.2% | **1.37 (1.23, 1.51)** | **1.34 (1.21, 1.49)** | | -0.28 | | 0.49 | **-0.08 (-0.11, -0.05)** | | **-0.07 (-0.10, -0.04)** |
| Access to improved water | |  |  |  |  | |  | |  |  | |  |
|  | No | 857 | 23.1% | Reference | Reference | | -0.25 | | 0.51 | Reference | | Reference |
|  | Yes | 692 | 23.0% | 0.99 (0.97, 1.02) | 1.04 (0.90, 1.21) | | -0.24 | | 0.49 | 0.01 (-0.01, 0.04) | | **-0.04 (-0.07, -0.01)** |
| Improved defecation facility | |  |  |  |  | |  | |  |  | |  |
|  | No | 1260 | 23.2% | Reference | Reference | | -0.26 | | 0.50 | Reference | | Reference |
|  | Yes | 289 | 22.5% | 0.96 (0.83, 1.11) | 0.89 (0.74, 1.06) | | -0.21 | | 0.49 | **0.05 (0.01, 0.08)** | | **0.07 (0.03, 0.11)** |
| Wealth index | |  |  |  |  | |  | |  |  | |  |
|  | Lowest quintile | 403 | 28.1% | Reference | Reference | | -0.25 | | 0.49 | Reference | | Reference |
|  | Second lowest | 443 | 31.2% | 1.11 (0.99, 1.24) | 1.06 (0.92, 1.21) | | -0.29 | | 0.52 | -0.03 (-0.07, 0.01) | | -0.01 (-0.05, 0.03) |
|  | Middle | 435 | 28.6% | 1.02 (0.91, 1.14) | 0.96 (0.83, 1.11) | | -0.24 | | 0.50 | 0.05 (-0.03, 0.04) | | 0.03 (-0.01, 0.07) |
|  | Second highest | 330 | 25.2% | 0.89 (0.79, 1.02) | 0.92 (0.80, 1.06) | | -0.23 | | 0.47 | 0.03 (-0.01, 0.07) | | 0.04 (-0.001, 0.08) |
|  | Highest quintile | 382 | 28.0% | 0.99 (0.89, 1.12) | 0.93 (0.80, 1.07) | | -0.22 | | 0.52 | 0.03 (-0.005, 0.07) | | **0.0.08 (0.04, 0.12)** |

1. Flush toilet, ventilated improved pit latrine with or without water seal, or pour flush toilet not shared with other households [↑](#endnote-ref-1)
2. Visible blood in stool observed by study staff or reported by caregiver at presentation; discharge diagnosis of dysentery per managing clinician upon leaving the healthcare facility; or observed in stool sample by laboratory staff [↑](#endnote-ref-2)
3. Per discharge diagnoses documented on medical records [↑](#endnote-ref-3)
4. Indicates number with severe linear growth faltering [↑](#endnote-ref-4)
5. Data on duration of diarrhea for the 7 days before enrollment were ascertained at enrollment (children with diarrhea lasting longer than 7 days were excluded at this point), and data on diarrhea duration for the 14 days following enrollment were ascertained with a memory aid suitable for groups of all literacy levels, which the caregiver returned at the 60-day follow up visit [↑](#endnote-ref-5)
